# Syntheses and crystal structures of three novel oxalate coordination compounds: Rb_2_Co(C_2_O_4_)_2_·4H_2_O, Rb_2_CoCl_2_(C_2_O_4_) and K_2_Li_2_Cu(C_2_O_4_)_3_·2H_2_O

**DOI:** 10.1107/S2056989023001822

**Published:** 2023-03-07

**Authors:** Rebecca Clulow, Philip Lightfoot

**Affiliations:** a Department of Chemistry - Ångström Laboratory, Lägerhyddsvägen 1, Box 538, 751 21, Uppsala, Sweden; b School of Chemistry, University of St Andrews, KY16 9ST, Scotland, United Kingdom; Vienna University of Technology, Austria

**Keywords:** crystal structure, oxalates, coordination compounds, first-row transition metals

## Abstract

Single crystals of three novel transition-metal oxalates, dirubidium di­aqua­dioxalatocobalt(II) dihydrate or dirubidium cobalt(II) bis­(oxalate) tetra­hydrate, Rb_2_[Co(C_2_O_4_)_2_(H_2_O)_2_]·2H_2_O, (I), *catena*-poly[dirubidium [[di­chlorido­cobalt(II)]-μ-oxalato]] or dirubidium cobalt(II) dichloride oxalate, {Rb_2_[CoCl_2_(C_2_O_4_)]}_*n*_, (II), and poly[dipotassium [tri-μ-oxalato-copper(II)dilithium] dihydrate] or dipotassium dilithium copper(II) tris­(oxalate) dihydrate, {K_2_[Li_2_Cu(C_2_O_4_)_3_]·2H_2_O}_*n*_, (III), have been grown under hydro­thermal conditions and their crystal structures determined using single-crystal X-ray diffraction. The structure of (I) exhibits isolated octa­hedral [Co(C_2_O_4_)_2_(H_2_O)_2_] units, whereas (II) consists of *trans* chains of Co^2+^ ions bridged by bidentate oxalato ligands and (III) displays a novel tri-periodic network of Li^+^ and Cu^2+^ ions linked by oxalato bridging ligands.

## Abstract 2

Rb_2_Co(C_2_O_4_)_2_·4H_2_O consists of isolated [Co(C_2_O_4_)_2_·2H_2_O] octa­hedra which are connected only by hydrogen bonding of the water mol­ecules. Rb_2_CoCl_2_(C_2_O_4_) consists of chains of Co^2+^ cations connected *via* oxalate bridging ligands. In K_2_Li_2_Cu(C_2_O_4_)_3_·2H_2_O, the metal cations are inter­connected by oxalate ligands forming a novel three-periodic network.

## 1. Chemical context

Oxalate-based transition-metal complexes have long attracted inter­est because of their promising magnetic and electrochemical properties. Their magnetic properties are in part due to the oxalato ligand, which is known to facilitate magnetic exchange between transition-metal cations, and the compounds are known to exhibit both ferro- and anti­ferromagnetic inter­actions (Miller & Drillon, 2002 ▸; Baran, 2014 ▸). In addition to their magnetic properties, there have also been numerous studies concerning their electrochemical properties, which have shown promising results (Pramanik *et al.*, 2022 ▸; Cai *et al.*, 2020 ▸; Yao *et al.*, 2019 ▸). Part of the appeal of oxalate-based coordination compounds is due to their high degree of structural diversity, as a result of the oxalate ligand, which can adopt 17 different coordination modes and act as a mono-, bi-, tri- or tetra­dentate ligand (Rao *et al.*, 2004 ▸). This has led to a vast compositional area, which is yet to be fully explored. In this context, the crystal structures of three new oxalate-based coordination compounds are reported and discussed herein.

## 2. Structural commentary

**Figure 1:**
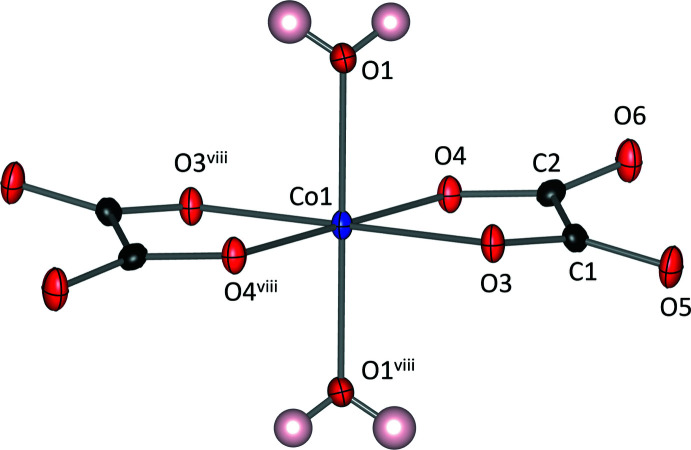
Coordination environment of Co^2+^ in Rb_2_Co(C_2_O_4_)_2_·4H_2_O (I). Colour code: Co (blue), C (black), O (red) and H (light pink). Displacement ellipsoids are drawn at the 50% probability level. [Symmetry code: (viii) −*x* + 1, −*y*, −*z* + 1].

**Figure 2:**
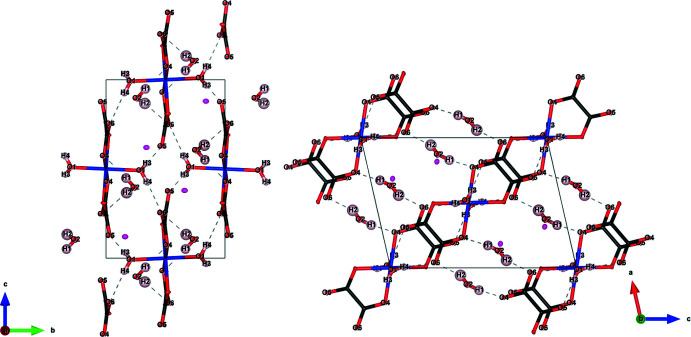
The hydrogen-bonding network of Rb_2_Co(C_2_O_4_)_2_·2H_2_O (I) viewed along the *a* and *b* axes. Displacement ellipsoids are drawn at the 30% probability level. The hydrogen bonds are shown as dashed lines. Colour code: Rb (pink), Co (blue), C (black), O (red) and H (light pink).

Rb_2_Co(C_2_O_4_)_2_·4H_2_O (I) consists of isolated [Co(C_2_O_4_)_2_(H_2_O)_2_] octa­hedra. The Co^2+^ cation lies on the 2*c* Wyckoff position with a site symmetry of 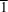 , leading to a *trans* disposition of the bidentate oxalato and aqua ligands (Fig. 1 ▸). The average Co—O bond length was determined as 2.080 Å, with a calculated bond-valence sum of 2.10 valence units. The Rb^+^ cation has a coordination number of 11, defined by oxalate O atoms and water mol­ecules. While the water mol­ecule involving O1 coordinates to both Rb^+^ and Co^2+^, the second water mol­ecule involving O2 solely bonds to the alkali metal cation. The [Co(C_2_O_4_)_2_(H_2_O)_2_] octa­hedra are inter­linked by hydrogen bonding of both types of water mol­ecules, as shown in Fig. 2 ▸. The mutually *trans* coordinating water mol­ecules (H3, O1, H4) form hydrogen bonds with the oxalate ligands of the neighbouring [Co(C_2_O_4_)_2_(H_2_O)_2_] octa­hedra, whilst the second type of water mol­ecule (H1, O2, H2) forms hydrogen bonds (in part bifurcated) with the oxalate ligands of two separate [Co(C_2_O_4_)_2_(H_2_O)_2_] octa­hedra. Numerical data for the hydrogen-bonding inter­actions are given in Table 1 ▸.

**Figure 3:**
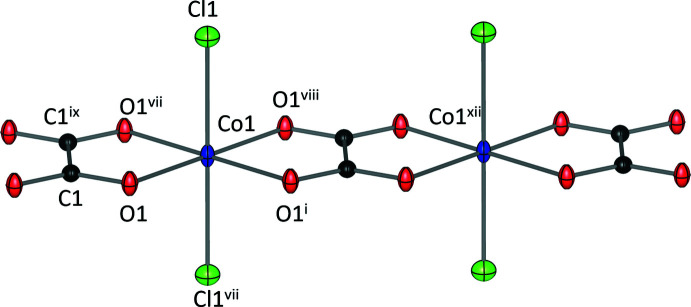
Coordination environment of Co^2+^ in Rb_2_CoCl_2_(C_2_O_4_) (II). Colour code: Co (blue), Cl (green), C (black) and O (red). Displacement ellipsoids are drawn at 50% probability level. [Symmetry codes: (i) −*x* + 1, *y*, −*z* + 1; (vii) *x*, −*y* + 2, *z*; (viii) −*x* + 1, −*y* + 2, −*z* + 1; (ix) −*x* + 2, −*y* + 2, −*z* + 1; (xii) *x* − 1, *y*, *z*].

**Figure 4:**
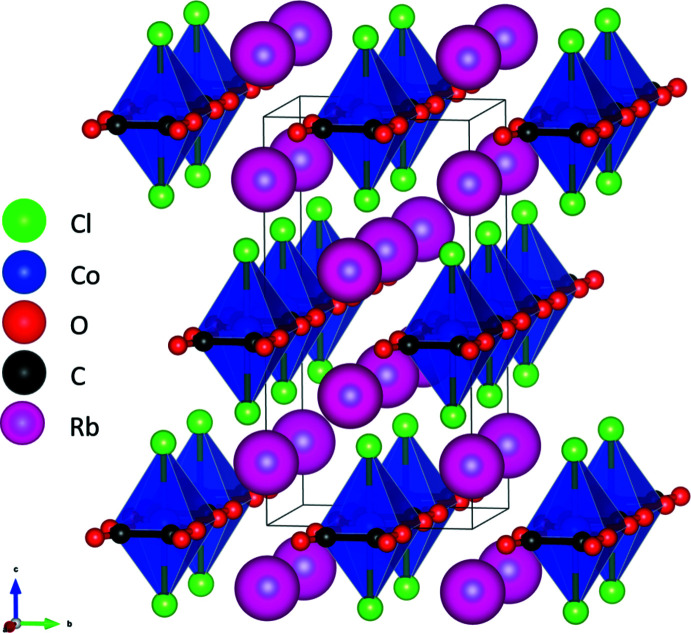
The crystal structure of Rb_2_CoCl_2_(C_2_O_4_) (II) in a view approximately along the *a* axis.

Rb_2_CoCl_2_(C_2_O_4_) (II) consists of octa­hedrally coordinated Co^2+^ cations. They are linked by bis-bidentate oxalate ligands to form chains extending parallel to the *a* axis, as shown in Fig. 3 ▸. The oxalate ligands are mutually *trans* to one another whilst the Cl^−^ anions cap each side of the octa­hedron. Co—O bond lengths are 2.0616 (17) Å and longer for the Co—Cl bond at 2.4863 (9) Å, with a calculated bond-valence sum of 2.03 valence units for Co. The Rb^+^ cation has a coordination number of eight and lies between the layers formed by the Co^2+^ chains (Fig. 4 ▸), with no direct connectivity between the chains. Each of the atoms lies on a special position within the unit cell with Wyckoff positions/site symmetries: Rb^+^ (4*i*, *mm*2), Co^2+^ (2*d*, *mmm*), Cl^−^ (4*j*, *mm*2), O (8*n*,. .*m*) and C (4*h*, *m*2*m*). The presence of the oxalate-bridged Co^2+^ chain could allow for magnetic exchange (García-Couceiro *et al.*, 2004 ▸), hence the magnetic properties of the compound should also be investigated in the future.

**Figure 5:**
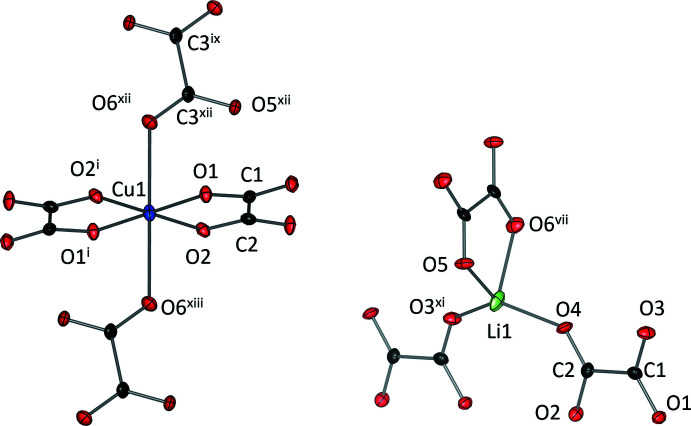
Coordination environments of Cu^2+^ and of Li^+^ in the crystal structure of K_2_Li_2_Cu(C_2_O_4_)_3_·2H_2_O (III). Colour code: Li (green), Cu (Blue), C (black) and O (red). Displacement ellipsoids are drawn at the 50% probability level. [Symmetry codes: (i) −*x* + 2, −*y* + 2, −*z* + 2; (vii) −*x*, −*y*, −*z* + 1; (ix) *x* + 1, *y* + 1, *z*; (xi) *x* − 1, *y*, *z*; (xii) −*x* + 1, −*y* + 1, −*z* + 1; (xiii) *x* + 1, *y* + 1, *z* + 1].

**Figure 6:**
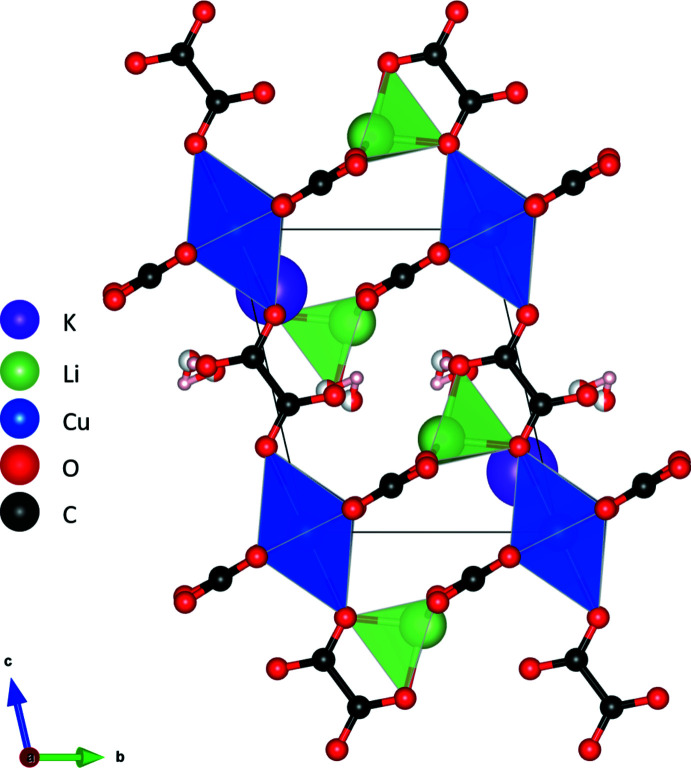
The crystal structure of K_2_Li_2_Cu(C_2_O_4_)_3_·2H_2_O (III) viewed along the *a* axis.

The Cu^2+^ and Li^+^ binding environments of K_2_Li_2_Cu(C_2_O_4_)_3_·2H_2_O (III) are shown in Fig. 5 ▸. The *d* ^9^ Cu^2+^ cations display classic Jahn–Teller distortion with elongation of the axial Cu—O bonds. The equatorial Cu—O bond lengths are 1.938 (3) (O2) and 1.942 (3) (O1) Å whilst the axial bonds are significantly longer at 2.473 (4) Å (O6). The Cu^2+^ ion lies on a special position with Wyckoff position and site symmetry of 6*b* and 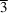 , respectively. The Cu^2+^ coordination environment consists of four oxalate ligands, two of which act as bidentate bridging ligands and two of which are axially oriented and bind to four metal cations with a tricoordinate oxygen atom. The Li^+^ cation is tetra­hedrally coordinated by three oxalate mol­ecules, one of which is bidentate whilst the other two are monodentate. The Cu^2+^ and Li^+^-centred polyhedra are inter­connected into a tri-periodic network, as shown in Fig. 6 ▸. The coordination environment of the K^+^ cation lies within this network and consists of eight oxygen atoms from the oxalate ligands and two water mol­ecules. These water mol­ecules exhibit disorder of the O7 atom, which is split into two positions. The inter­atomic distances between the water mol­ecules is ∼3.7 Å, which is too far apart to facilitate hydrogen bonding.

## 3. Database survey

Database surveys were carried out using the Cambridge Structural Database (CSD, last update November 2022; Groom *et al.*, 2016 ▸) for compounds with structural similarities to the three new oxalate coordination compounds reported here. For (I), a search for first-row transition metals with the same coordination environment produced numerous results for a range of transition metals. The most similar is DIHXID [dipotassium bis­(oxalato)di­aqua­cobalt(II) tetra­hydrate; Chylewska *et al.*, 2013 ▸), which has the same formula type and coordination environment as (I) although with K^+^ rather Rb^+^ cations, but is not isostructural. For (II), there are several compounds containing transition-metal oxalate chains with the same binding environment, although with quite different cations involved. For example BEJHOQ {*catena*-[bis­(2-(5,6-di­hydro-2*H*-[1,3]di­thiolo[4,5-*b*][1,4]dithiin-2-yl­idene)-5,6-di­hydro-2*H*-[1,3]di­thiolo[4,5-*b*][1,4]dithiin-1-ium) bis­(μ-oxalato)tetra­chloro­diiron(III) di­chloro­methane solvate]} and EYALIB {*catena*-[bis­(2-(5,6-di­hydro-2*H*-[1,3]di­selenolo[4,5-*b*][1,4]dithiin-2-yl­idene)-5,6-di­hydro-2*H*-[1,3]di­selenolo[4,5-*b*][1,4]dithiin-1-ium) bis­(μ-oxalato)tetra­chloro­diiron(III)]; Zhang, 2016 ▸, 2017 ▸). The database survey of compounds with similar binding environments to (III) focused on first-row transition metals with two bidentate and two mutually *trans* monodentate oxalate ligands, containing a tricoordinating oxygen atom. The search revealed evidence of only two similar compounds, *viz*. ADAJUL [octa­ammonium hexa­kis­(μ_2_-oxalato-*O*,*O*,*O*′)bis­(oxalato-*O*,*O*′)di­aqua­tetra­copper(II) tetra­hydrate] and ASOXOV {bis­[1,4-diazo­niabi­cyclo­(2.2.2)octa­ne]bis­(μ_2_-oxalato)di­aqua­bis­(oxalato)dicopper(II) tetra­hydrate; Kadir *et al.*, 2006 ▸; Keene *et al.*, 2004 ▸}. These contain similar types of linkages, although with only one type of cation and only as discrete mol­ecules rather than coordination polymers. Hence, (III) represents the first example of this type of binding environment.

## 4. Synthesis and crystallization

The samples were synthesized *via* hydro­thermal syntheses in the temperature range 433–463 K over four days, from commercially available starting reagents. Compounds (I) and (II) were synthesized as by-products from the reaction of rubidium carbonate, sodium carbonate, cobalt chloride hexa­hydrate and oxalic acid dihydrate in molar ratios of 2:2:1:1.5 and 1:1.5:1:1.5 at 433 and 463 K, respectively. Compound (III) was synthesized by the reaction of potassium carbonate, lithium carbonate, copper chloride dihydrate and oxalic acid dihydrate (1:3:1:3) at 463 K. Single crystals were isolated from a mixture of products for further analysis. The resulting crystals were filtered and dried overnight at 323 K prior to analysis by X-ray diffraction.

## 5. Refinement

Crystal data and refinement details of the three compounds are summarized in Table 2 ▸. The H atoms in (I) and (III) were allowed to refine freely. The disordered oxygen atom in compound III (O7) was split over two positions with their occupancies fixed at 0.5 while their atomic coordinates and *U* ^ij^s were refined independently.

## Supplementary Material

Crystal structure: contains datablock(s) I, II, III, global. DOI: 10.1107/S2056989023001822/wm5668sup1.cif

Structure factors: contains datablock(s) I. DOI: 10.1107/S2056989023001822/wm5668Isup2.hkl

Structure factors: contains datablock(s) II. DOI: 10.1107/S2056989023001822/wm5668IIsup3.hkl

Structure factors: contains datablock(s) III. DOI: 10.1107/S2056989023001822/wm5668IIIsup4.hkl

CCDC references: 2245039, 2245038, 2245037

Additional supporting information:  crystallographic information; 3D view; checkCIF report

## Figures and Tables

**Table 1:** Hydrogen-bond geometry (Å, °) for (I)

*D*—H⋯*A*	*D*—H	H⋯*A*	*D*⋯*A*	*D*—H⋯*A*
O2—H1⋯O4	0.89 (4)	2.00 (4)	2.880 (2)	171 (4)
O2—H2⋯O4^i^	0.85 (5)	2.47 (5)	3.187 (2)	143 (4)
O2—H2⋯O6^i^	0.85 (5)	2.20 (5)	3.008 (2)	159 (4)
O1—H3⋯O5^ii^	0.76 (3)	1.98 (3)	2.736 (2)	174 (3)
O1—H4⋯O6^iii^	0.78 (3)	2.05 (3)	2.825 (2)	172 (3)

Symmetry codes: (i) 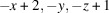 ; (ii) 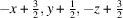 ; (iii) 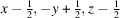 .

**Table 2:** Experimental details

	(I)	(II)	(III)
Crystal data			
Chemical formula	Rb_2_[Co(C_2_O_4_)_2_(H_2_O)_2_]·2H_2_O	Rb_2_[CoCl_2_(C_2_O_4_)]	K_2_[Li_2_Cu(C_2_O_4_)_3_]·2H_2_O
*M*_r_	477.97	388.79	455.71
Crystal system, space group	Monoclinic, *P*2_1_/*n*	Orthorhombic, *I* *m* *m* *m*	Triclinic, *P* 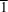
Temperature (K)	173	173	173
*a*, *b*, *c* (Å)	7.8434 (5), 7.0795 (4), 10.9133 (7)	5.3445 (3), 6.4380 (4), 12.5866 (8)	6.1847 (4), 7.2575 (5), 8.1795 (5)
α, β, γ (°)	90, 102.836 (8), 90	90, 90, 90	101.327 (11), 91.723 (11), 113.563 (11)
*V* (Å^3^)	590.84 (7)	433.08 (5)	327.56 (5)
*Z*	2	2	1
Radiation type	Mo *K*α	Mo *K*α	Mo *K*α
μ (mm^−1^)	9.70	13.73	2.39
Crystal size (mm)	0.21 × 0.16 × 0.08	0.20 × 0.15 × 0.07	0.14 × 0.14 × 0.07

Data collection			
Diffractometer	Rigaku Mercury2 (2x2 bin mode)	Rigaku Mercury2 (2x2 bin mode)	Rigaku Mercury2 (2x2 bin mode)
Absorption correction	Multi-scan (*SADABS*; Krause *et al.*, 2015 ▸)	Multi-scan (*SADABS*; Krause *et al.*, 2015 ▸)	Multi-scan (*SADABS*; Krause *et al.*, 2015 ▸)
*T*_min_, *T*_max_	0.681, 1.00	0.671, 1.00	0.610, 1.00
No. of measured, independent and observed [*I* > 2σ(*I*)] reflections	5799, 1343, 1169	2218, 310, 296	3403, 1500, 1077
*R*_int_	0.039	0.034	0.095
(sin θ/λ)_max_ (Å^−1^)	0.650	0.649	0.651

Refinement			
*R*[*F* ^2^ > 2σ(*F* ^2^)], *wR*(*F* ^2^), *S*	0.021, 0.050, 0.97	0.018, 0.043, 1.11	0.045, 0.114, 0.94
No. of reflections	1343	310	1500
No. of parameters	104	22	132
H-atom treatment	All H-atom parameters refined	–	All H-atom parameters refined
Δρ_max_, Δρ_min_ (e Å^−3^)	0.65, −0.64	0.63, −0.53	1.03, −1.01

Computer programs: *CrystalClear* (Rigaku, 2015 ▸), *SHELXT* (Sheldrick, 2015*a* ▸), *SHELXL* (Sheldrick, 2015*b* ▸) and *ORTEP* for Windows and *WinGX* (Farrugia, 2012 ▸).

## supplementary crystallographic information

### Dirubidium diaquadioxalatocobalt(II) dihydrate (I) Crystal data

**Table:**

Rb_2_[Co(C_2_O_4_)_2_(H_2_O)_2_]·2H_2_O	*F*(000) = 458
*M**_r_* = 477.97	*D*_x_ = 2.687 Mg m^−^^3^
Monoclinic, *P*2_1_/*n*	Mo *K*α radiation, λ = 0.71075 Å
Hall symbol: -P 2yn	Cell parameters from 935 reflections
*a* = 7.8434 (5) Å	θ = 1.9–27.5°
*b* = 7.0795 (4) Å	µ = 9.70 mm^−^^1^
*c* = 10.9133 (7) Å	*T* = 173 K
β = 102.836 (8)°	Prism, orange
*V* = 590.84 (7) Å^3^	0.21 × 0.16 × 0.08 mm
*Z* = 2	

### Dirubidium diaquadioxalatocobalt(II) dihydrate (I) Data collection

**Table:**

Rigaku Mercury2 (2x2 bin mode) diffractometer	1343 independent reflections
Radiation source: Sealed Tube	1169 reflections with *I* > 2σ(*I*)
Detector resolution: 13.6612 pixels mm^-1^	*R*_int_ = 0.039
profile data from ω–scans	θ_max_ = 27.5°, θ_min_ = 2.9°
Absorption correction: multi-scan (SADABS; Krause *et al.*, 2015)	*h* = −10→10
*T*_min_ = 0.681, *T*_max_ = 1.00	*k* = −9→9
5799 measured reflections	*l* = −14→13

### Dirubidium diaquadioxalatocobalt(II) dihydrate (I) Refinement

**Table:**

Refinement on *F*^2^	0 restraints
Least-squares matrix: full	Hydrogen site location: difference Fourier map
*R*[*F*^2^ > 2σ(*F*^2^)] = 0.021	All H-atom parameters refined
*wR*(*F*^2^) = 0.050	*w* = 1/[σ^2^(*F*_o_^2^) + (0.0323*P*)^2^] where *P* = (*F*_o_^2^ + 2*F*_c_^2^)/3
*S* = 0.97	(Δ/σ)_max_ < 0.001
1343 reflections	Δρ_max_ = 0.65 e Å^−^^3^
104 parameters	Δρ_min_ = −0.64 e Å^−^^3^

### Dirubidium diaquadioxalatocobalt(II) dihydrate (I) Special details

**Table:**

Geometry. All esds (except the esd in the dihedral angle between two l.s. planes) are estimated using the full covariance matrix. The cell esds are taken into account individually in the estimation of esds in distances, angles and torsion angles; correlations between esds in cell parameters are only used when they are defined by crystal symmetry. An approximate (isotropic) treatment of cell esds is used for estimating esds involving l.s. planes.

### Dirubidium diaquadioxalatocobalt(II) dihydrate (I) Fractional atomic coordinates and isotropic or equivalent isotropic displacement parameters (Å^2^)

**Table:**

	*x*	*y*	*z*	*U*_iso_*/*U*_eq_	
Rb1	0.81534 (3)	0.66030 (3)	0.37962 (2)	0.01935 (9)	
Co1	0.500000	0.000000	0.500000	0.01014 (11)	
O1	0.5165 (2)	0.2912 (2)	0.49112 (17)	0.0160 (3)	
O2	0.8705 (2)	0.2408 (3)	0.39553 (17)	0.0236 (4)	
O3	0.50631 (18)	0.0198 (2)	0.69060 (13)	0.0143 (3)	
O4	0.77067 (18)	−0.0152 (2)	0.57235 (13)	0.0139 (3)	
O5	0.6916 (2)	−0.0314 (2)	0.87422 (14)	0.0186 (3)	
O6	0.96271 (19)	0.0330 (2)	0.75326 (14)	0.0191 (3)	
C1	0.6578 (3)	−0.0014 (3)	0.75938 (19)	0.0118 (4)	
C2	0.8121 (3)	0.0076 (3)	0.69128 (19)	0.0122 (4)	
H1	0.828 (5)	0.168 (5)	0.449 (4)	0.056 (11)*	
H2	0.942 (6)	0.173 (6)	0.367 (4)	0.079 (15)*	
H3	0.594 (4)	0.342 (4)	0.532 (3)	0.023 (8)*	
H4	0.494 (4)	0.345 (4)	0.427 (3)	0.029 (9)*	

### Dirubidium diaquadioxalatocobalt(II) dihydrate (I) Atomic displacement parameters (Å^2^)

**Table:**

	*U*^11^	*U*^22^	*U*^33^	*U*^12^	*U*^13^	*U*^23^
Rb1	0.01880 (14)	0.02412 (14)	0.01580 (13)	0.00190 (8)	0.00529 (9)	0.00010 (8)
Co1	0.0105 (2)	0.01303 (19)	0.00658 (19)	0.00024 (14)	0.00112 (15)	−0.00043 (14)
O1	0.0195 (9)	0.0142 (7)	0.0118 (8)	−0.0033 (6)	−0.0020 (7)	0.0004 (6)
O2	0.0248 (10)	0.0268 (9)	0.0231 (9)	−0.0002 (8)	0.0138 (8)	0.0007 (7)
O3	0.0123 (7)	0.0216 (7)	0.0083 (7)	0.0009 (6)	0.0011 (6)	−0.0011 (6)
O4	0.0121 (7)	0.0202 (7)	0.0093 (7)	0.0010 (6)	0.0024 (6)	−0.0012 (6)
O5	0.0180 (8)	0.0291 (8)	0.0081 (7)	0.0000 (6)	0.0016 (6)	0.0021 (6)
O6	0.0126 (8)	0.0305 (9)	0.0126 (7)	−0.0007 (7)	−0.0009 (6)	0.0005 (7)
C1	0.0138 (10)	0.0119 (9)	0.0099 (9)	−0.0008 (7)	0.0031 (8)	−0.0019 (8)
C2	0.0134 (10)	0.0103 (9)	0.0130 (10)	0.0020 (8)	0.0032 (8)	0.0014 (8)

### Dirubidium diaquadioxalatocobalt(II) dihydrate (I) Geometric parameters (Å, º)

**Table:**

Rb1—O2	3.0003 (18)	Co1—O1^viii^	2.0690 (16)
Rb1—O5^i^	3.0209 (16)	Co1—O1	2.0690 (16)
Rb1—O3^ii^	3.0819 (15)	Co1—O3^viii^	2.0744 (14)
Rb1—O2^iii^	3.0856 (19)	Co1—O3	2.0744 (14)
Rb1—O5^ii^	3.1023 (16)	Co1—O4	2.0969 (14)
Rb1—O6^iv^	3.1190 (15)	Co1—O4^viii^	2.0969 (14)
Rb1—O2^v^	3.1461 (19)	O3—C1	1.265 (2)
Rb1—O4^vi^	3.1862 (15)	O4—C2	1.276 (3)
Rb1—O1^vii^	3.2421 (17)	O5—C1	1.240 (3)
Rb1—O6^v^	3.3122 (16)	O6—C2	1.237 (3)
Rb1—O3^vii^	3.3497 (15)	C1—C2	1.556 (3)
			
O2—Rb1—O5^i^	62.28 (5)	O2^iii^—Rb1—O3^vii^	58.91 (4)
O2—Rb1—O3^ii^	62.87 (4)	O5^ii^—Rb1—O3^vii^	149.98 (4)
O5^i^—Rb1—O3^ii^	121.12 (4)	O6^iv^—Rb1—O3^vii^	69.30 (4)
O2—Rb1—O2^iii^	105.67 (5)	O2^v^—Rb1—O3^vii^	116.60 (4)
O5^i^—Rb1—O2^iii^	148.26 (5)	O4^vi^—Rb1—O3^vii^	58.39 (4)
O3^ii^—Rb1—O2^iii^	67.65 (4)	O1^vii^—Rb1—O3^vii^	52.51 (4)
O2—Rb1—O5^ii^	65.45 (5)	O6^v^—Rb1—O3^vii^	84.20 (4)
O5^i^—Rb1—O5^ii^	95.16 (4)	O1^viii^—Co1—O1	180.0
O3^ii^—Rb1—O5^ii^	42.24 (4)	O1^viii^—Co1—O3^viii^	89.52 (6)
O2^iii^—Rb1—O5^ii^	106.35 (5)	O1—Co1—O3^viii^	90.48 (6)
O2—Rb1—O6^iv^	72.16 (5)	O1^viii^—Co1—O3	90.48 (6)
O5^i^—Rb1—O6^iv^	90.38 (4)	O1—Co1—O3	89.52 (6)
O3^ii^—Rb1—O6^iv^	92.16 (4)	O3^viii^—Co1—O3	180.0
O2^iii^—Rb1—O6^iv^	58.00 (5)	O1^viii^—Co1—O4	89.98 (6)
O5^ii^—Rb1—O6^iv^	128.06 (4)	O1—Co1—O4	90.02 (6)
O2—Rb1—O2^v^	95.58 (5)	O3^viii^—Co1—O4	99.83 (6)
O5^i^—Rb1—O2^v^	64.67 (5)	O3—Co1—O4	80.17 (6)
O3^ii^—Rb1—O2^v^	101.60 (4)	O1^viii^—Co1—O4^viii^	90.02 (6)
O2^iii^—Rb1—O2^v^	146.79 (5)	O1—Co1—O4^viii^	89.98 (6)
O5^ii^—Rb1—O2^v^	59.77 (4)	O3^viii^—Co1—O4^viii^	80.17 (6)
O6^iv^—Rb1—O2^v^	155.02 (4)	O3—Co1—O4^viii^	99.83 (6)
O2—Rb1—O4^vi^	135.32 (4)	O4—Co1—O4^viii^	180.0
O5^i^—Rb1—O4^vi^	73.17 (4)	Co1—O1—Rb1^vii^	91.19 (6)
O3^ii^—Rb1—O4^vi^	151.80 (4)	Rb1—O2—Rb1^ix^	95.49 (5)
O2^iii^—Rb1—O4^vi^	114.32 (4)	Rb1—O2—Rb1^v^	84.42 (4)
O5^ii^—Rb1—O4^vi^	117.99 (4)	Rb1^ix^—O2—Rb1^v^	156.77 (7)
O6^iv^—Rb1—O4^vi^	113.04 (4)	C1—O3—Co1	113.38 (13)
O2^v^—Rb1—O4^vi^	60.44 (4)	C1—O3—Rb1^x^	94.92 (12)
O2—Rb1—O1^vii^	101.48 (5)	Co1—O3—Rb1^x^	137.42 (6)
O5^i^—Rb1—O1^vii^	59.06 (4)	C1—O3—Rb1^vii^	140.90 (12)
O3^ii^—Rb1—O1^vii^	153.07 (4)	Co1—O3—Rb1^vii^	88.13 (5)
O2^iii^—Rb1—O1^vii^	98.78 (5)	Rb1^x^—O3—Rb1^vii^	88.83 (4)
O5^ii^—Rb1—O1^vii^	153.95 (4)	C2—O4—Co1	112.61 (13)
O6^iv^—Rb1—O1^vii^	61.33 (4)	C2—O4—Rb1^xi^	136.21 (12)
O2^v^—Rb1—O1^vii^	101.68 (5)	Co1—O4—Rb1^xi^	92.24 (5)
O4^vi^—Rb1—O1^vii^	54.53 (4)	C1—O5—Rb1^xii^	141.22 (13)
O2—Rb1—O6^v^	126.25 (5)	C1—O5—Rb1^x^	94.51 (12)
O5^i^—Rb1—O6^v^	143.03 (4)	Rb1^xii^—O5—Rb1^x^	84.84 (4)
O3^ii^—Rb1—O6^v^	66.26 (4)	C2—O6—Rb1^xiii^	145.41 (13)
O2^iii^—Rb1—O6^v^	68.49 (4)	C2—O6—Rb1^v^	112.75 (13)
O5^ii^—Rb1—O6^v^	65.79 (4)	Rb1^xiii^—O6—Rb1^v^	88.88 (4)
O6^iv^—Rb1—O6^v^	126.49 (2)	O5—C1—O3	125.57 (19)
O2^v^—Rb1—O6^v^	78.39 (4)	O5—C1—C2	118.35 (18)
O4^vi^—Rb1—O6^v^	87.81 (4)	O3—C1—C2	116.07 (17)
O1^vii^—Rb1—O6^v^	132.20 (4)	O6—C2—O4	124.8 (2)
O2—Rb1—O3^vii^	140.70 (5)	O6—C2—C1	119.63 (18)
O5^i^—Rb1—O3^vii^	110.22 (4)	O4—C2—C1	115.52 (18)
O3^ii^—Rb1—O3^vii^	125.45 (2)		

Symmetry codes: (i) −*x*+3/2, *y*+1/2, −*z*+3/2; (ii) *x*+1/2, −*y*+1/2, *z*−1/2; (iii) −*x*+3/2, *y*+1/2, −*z*+1/2; (iv) *x*−1/2, −*y*+1/2, *z*−1/2; (v) −*x*+2, −*y*+1, −*z*+1; (vi) *x*, *y*+1, *z*; (vii) −*x*+1, −*y*+1, −*z*+1; (viii) −*x*+1, −*y*, −*z*+1; (ix) −*x*+3/2, *y*−1/2, −*z*+1/2; (x) *x*−1/2, −*y*+1/2, *z*+1/2; (xi) *x*, *y*−1, *z*; (xii) −*x*+3/2, *y*−1/2, −*z*+3/2; (xiii) *x*+1/2, −*y*+1/2, *z*+1/2.

### Dirubidium diaquadioxalatocobalt(II) dihydrate (I) Hydrogen-bond geometry (Å, º)

**Table:**

*D*—H···*A*	*D*—H	H···*A*	*D*···*A*	*D*—H···*A*
O2—H1···O4	0.89 (4)	2.00 (4)	2.880 (2)	171 (4)
O2—H2···O4^xiv^	0.85 (5)	2.47 (5)	3.187 (2)	143 (4)
O2—H2···O6^xiv^	0.85 (5)	2.20 (5)	3.008 (2)	159 (4)
O1—H3···O5^i^	0.76 (3)	1.98 (3)	2.736 (2)	174 (3)
O1—H4···O6^iv^	0.78 (3)	2.05 (3)	2.825 (2)	172 (3)

Symmetry codes: (i) −*x*+3/2, *y*+1/2, −*z*+3/2; (iv) *x*−1/2, −*y*+1/2, *z*−1/2; (xiv) −*x*+2, −*y*, −*z*+1.

### catena-Poly[dirubidium [[dichloridocobalt(II)]-µ-oxalato]] (II) Crystal data

**Table:**

Rb_2_[CoCl_2_(C_2_O_4_)]	*F*(000) = 358
*M**_r_* = 388.79	*D*_x_ = 2.981 Mg m^−^^3^
Orthorhombic, *I**m**m**m*	Mo *K*α radiation, λ = 0.71075 Å
Hall symbol: -I 2 2	Cell parameters from 800 reflections
*a* = 5.3445 (3) Å	θ = 3.2–27.5°
*b* = 6.4380 (4) Å	µ = 13.73 mm^−^^1^
*c* = 12.5866 (8) Å	*T* = 173 K
*V* = 433.08 (5) Å^3^	Prism, purple
*Z* = 2	0.20 × 0.15 × 0.07 mm

### catena-Poly[dirubidium [[dichloridocobalt(II)]-µ-oxalato]] (II) Data collection

**Table:**

Rigaku Mercury2 (2x2 bin mode) diffractometer	310 independent reflections
Radiation source: Sealed Tube	296 reflections with *I* > 2σ(*I*)
Detector resolution: 13.6612 pixels mm^-1^	*R*_int_ = 0.034
profile data from ω–scans	θ_max_ = 27.5°, θ_min_ = 3.2°
Absorption correction: multi-scan (SADABS; Krause *et al.*, 2015)	*h* = −6→6
*T*_min_ = 0.671, *T*_max_ = 1.00	*k* = −8→8
2218 measured reflections	*l* = −16→16

### catena-Poly[dirubidium [[dichloridocobalt(II)]-µ-oxalato]] (II) Refinement

**Table:**

Refinement on *F*^2^	22 parameters
Least-squares matrix: full	0 restraints
*R*[*F*^2^ > 2σ(*F*^2^)] = 0.018	*w* = 1/[σ^2^(*F*_o_^2^) + (0.0276*P*)^2^] where *P* = (*F*_o_^2^ + 2*F*_c_^2^)/3
*wR*(*F*^2^) = 0.043	(Δ/σ)_max_ < 0.001
*S* = 1.11	Δρ_max_ = 0.63 e Å^−^^3^
310 reflections	Δρ_min_ = −0.53 e Å^−^^3^

### catena-Poly[dirubidium [[dichloridocobalt(II)]-µ-oxalato]] (II) Special details

**Table:**

Geometry. All esds (except the esd in the dihedral angle between two l.s. planes) are estimated using the full covariance matrix. The cell esds are taken into account individually in the estimation of esds in distances, angles and torsion angles; correlations between esds in cell parameters are only used when they are defined by crystal symmetry. An approximate (isotropic) treatment of cell esds is used for estimating esds involving l.s. planes.

### catena-Poly[dirubidium [[dichloridocobalt(II)]-µ-oxalato]] (II) Fractional atomic coordinates and isotropic or equivalent isotropic displacement parameters (Å^2^)

**Table:**

	*x*	*y*	*z*	*U*_iso_*/*U*_eq_	
Rb1	0.500000	0.500000	0.34642 (3)	0.01924 (15)	
Co1	0.500000	1.000000	0.500000	0.01332 (18)	
Cl1	0.500000	1.000000	0.30247 (7)	0.0198 (2)	
O1	0.7911 (3)	0.7898 (2)	0.500000	0.0154 (4)	
C1	1.000000	0.8775 (5)	0.500000	0.0123 (7)	

### catena-Poly[dirubidium [[dichloridocobalt(II)]-µ-oxalato]] (II) Atomic displacement parameters (Å^2^)

**Table:**

	*U*^11^	*U*^22^	*U*^33^	*U*^12^	*U*^13^	*U*^23^
Rb1	0.0230 (2)	0.0136 (2)	0.0211 (2)	0.000	0.000	0.000
Co1	0.0072 (3)	0.0105 (3)	0.0222 (3)	0.000	0.000	0.000
Cl1	0.0233 (4)	0.0172 (4)	0.0190 (4)	0.000	0.000	0.000
O1	0.0106 (9)	0.0106 (7)	0.0251 (8)	−0.0006 (6)	0.000	0.000
C1	0.0119 (16)	0.0125 (16)	0.0125 (14)	0.000	0.000	0.000

### catena-Poly[dirubidium [[dichloridocobalt(II)]-µ-oxalato]] (II) Geometric parameters (Å, º)

**Table:**

Rb1—O1	3.1045 (13)	Co1—O1^vii^	2.0616 (17)
Rb1—O1^i^	3.1045 (13)	Co1—O1^i^	2.0616 (17)
Rb1—O1^ii^	3.1045 (13)	Co1—O1^viii^	2.0616 (17)
Rb1—O1^iii^	3.1045 (13)	Co1—O1	2.0616 (17)
Rb1—Cl1^iv^	3.2639 (6)	Co1—Cl1^viii^	2.4863 (9)
Rb1—Cl1^v^	3.2639 (6)	Co1—Cl1	2.4863 (9)
Rb1—Cl1^vi^	3.2662 (3)	O1—C1	1.251 (2)
Rb1—Cl1	3.2662 (3)	C1—C1^ix^	1.577 (6)
			
O1—Rb1—O1^i^	60.14 (6)	O1^vii^—Co1—O1	82.03 (9)
O1—Rb1—O1^ii^	73.89 (5)	O1^i^—Co1—O1	97.97 (9)
O1^i^—Rb1—O1^ii^	102.98 (4)	O1^viii^—Co1—O1	180.0
O1—Rb1—O1^iii^	102.98 (4)	O1^vii^—Co1—Cl1^viii^	90.0
O1^i^—Rb1—O1^iii^	73.89 (5)	O1^i^—Co1—Cl1^viii^	90.0
O1^ii^—Rb1—O1^iii^	60.14 (6)	O1^viii^—Co1—Cl1^viii^	90.0
O1—Rb1—Cl1^iv^	140.15 (3)	O1—Co1—Cl1^viii^	90.0
O1^i^—Rb1—Cl1^iv^	86.98 (3)	O1^vii^—Co1—Cl1	90.0
O1^ii^—Rb1—Cl1^iv^	140.15 (3)	O1^i^—Co1—Cl1	90.0
O1^iii^—Rb1—Cl1^iv^	86.98 (3)	O1^viii^—Co1—Cl1	90.0
O1—Rb1—Cl1^v^	86.98 (3)	O1—Co1—Cl1	90.0
O1^i^—Rb1—Cl1^v^	140.15 (3)	Cl1^viii^—Co1—Cl1	180.0
O1^ii^—Rb1—Cl1^v^	86.98 (3)	Co1—Cl1—Rb1^iv^	125.042 (14)
O1^iii^—Rb1—Cl1^v^	140.15 (3)	Co1—Cl1—Rb1^v^	125.042 (14)
Cl1^iv^—Rb1—Cl1^v^	109.92 (3)	Rb1^iv^—Cl1—Rb1^v^	109.92 (3)
O1—Rb1—Cl1^vi^	134.25 (3)	Co1—Cl1—Rb1	80.247 (16)
O1^i^—Rb1—Cl1^vi^	134.25 (3)	Rb1^iv^—Cl1—Rb1	95.582 (8)
O1^ii^—Rb1—Cl1^vi^	60.86 (3)	Rb1^v^—Cl1—Rb1	95.582 (8)
O1^iii^—Rb1—Cl1^vi^	60.86 (3)	Co1—Cl1—Rb1^x^	80.246 (16)
Cl1^iv^—Rb1—Cl1^vi^	84.419 (8)	Rb1^iv^—Cl1—Rb1^x^	95.582 (8)
Cl1^v^—Rb1—Cl1^vi^	84.419 (8)	Rb1^v^—Cl1—Rb1^x^	95.582 (8)
O1—Rb1—Cl1	60.86 (3)	Rb1—Cl1—Rb1^x^	160.49 (3)
O1^i^—Rb1—Cl1	60.86 (3)	C1—O1—Co1	112.18 (16)
O1^ii^—Rb1—Cl1	134.25 (3)	C1—O1—Rb1^iii^	135.91 (8)
O1^iii^—Rb1—Cl1	134.25 (3)	Co1—O1—Rb1^iii^	90.94 (5)
Cl1^iv^—Rb1—Cl1	84.418 (8)	C1—O1—Rb1	135.91 (8)
Cl1^v^—Rb1—Cl1	84.418 (8)	Co1—O1—Rb1	90.94 (5)
Cl1^vi^—Rb1—Cl1	160.49 (3)	Rb1^iii^—O1—Rb1	77.02 (4)
O1^vii^—Co1—O1^i^	180.0	O1^xi^—C1—O1	126.4 (3)
O1^vii^—Co1—O1^viii^	97.97 (9)	O1^xi^—C1—C1^ix^	116.81 (15)
O1^i^—Co1—O1^viii^	82.03 (9)	O1—C1—C1^ix^	116.81 (15)

Symmetry codes: (i) −*x*+1, *y*, −*z*+1; (ii) *x*, −*y*+1, *z*; (iii) −*x*+1, −*y*+1, −*z*+1; (iv) −*x*+1/2, −*y*+3/2, −*z*+1/2; (v) −*x*+3/2, −*y*+3/2, −*z*+1/2; (vi) *x*, *y*−1, *z*; (vii) *x*, −*y*+2, *z*; (viii) −*x*+1, −*y*+2, −*z*+1; (ix) −*x*+2, −*y*+2, −*z*+1; (x) *x*, *y*+1, *z*; (xi) −*x*+2, *y*, −*z*+1.

### Poly[dipotassium [tri-µ-oxalatocopper(II)dilithium] dihydrate] (III) Crystal data

**Table:**

K_2_[Li_2_Cu(C_2_O_4_)_3_]·2H_2_O	*Z* = 1
*M**_r_* = 455.71	*F*(000) = 225
Triclinic, *P*1	*D*_x_ = 2.310 Mg m^−^^3^
Hall symbol: -P 1	Mo *K*α radiation, λ = 0.71075 Å
*a* = 6.1847 (4) Å	Cell parameters from 888 reflections
*b* = 7.2575 (5) Å	θ = 2.5–27.5°
*c* = 8.1795 (5) Å	µ = 2.38 mm^−^^1^
α = 101.327 (11)°	*T* = 173 K
β = 91.723 (11)°	Prism, blue
γ = 113.563 (11)°	0.14 × 0.14 × 0.07 mm
*V* = 327.56 (5) Å^3^	

### Poly[dipotassium [tri-µ-oxalatocopper(II)dilithium] dihydrate] (III) Data collection

**Table:**

Rigaku Mercury2 (2x2 bin mode) diffractometer	1500 independent reflections
Radiation source: Sealed Tube	1077 reflections with *I* > 2σ(*I*)
Detector resolution: 13.6612 pixels mm^-1^	*R*_int_ = 0.095
profile data from ω–scans	θ_max_ = 27.5°, θ_min_ = 2.6°
Absorption correction: multi-scan (SADABS; Krause *et al.*, 2015)	*h* = −8→8
*T*_min_ = 0.610, *T*_max_ = 1.00	*k* = −9→9
3403 measured reflections	*l* = −10→10

### Poly[dipotassium [tri-µ-oxalatocopper(II)dilithium] dihydrate] (III) Refinement

**Table:**

Refinement on *F*^2^	0 restraints
Least-squares matrix: full	Hydrogen site location: difference Fourier map
*R*[*F*^2^ > 2σ(*F*^2^)] = 0.045	All H-atom parameters refined
*wR*(*F*^2^) = 0.114	*w* = 1/[σ^2^(*F*_o_^2^) + (0.0439*P*)^2^] where *P* = (*F*_o_^2^ + 2*F*_c_^2^)/3
*S* = 0.94	(Δ/σ)_max_ < 0.001
1500 reflections	Δρ_max_ = 1.03 e Å^−^^3^
132 parameters	Δρ_min_ = −1.01 e Å^−^^3^

### Poly[dipotassium [tri-µ-oxalatocopper(II)dilithium] dihydrate] (III) Special details

**Table:**

Geometry. All esds (except the esd in the dihedral angle between two l.s. planes) are estimated using the full covariance matrix. The cell esds are taken into account individually in the estimation of esds in distances, angles and torsion angles; correlations between esds in cell parameters are only used when they are defined by crystal symmetry. An approximate (isotropic) treatment of cell esds is used for estimating esds involving l.s. planes.

### Poly[dipotassium [tri-µ-oxalatocopper(II)dilithium] dihydrate] (III) Fractional atomic coordinates and isotropic or equivalent isotropic displacement parameters (Å^2^)

**Table:**

	*x*	*y*	*z*	*U*_iso_*/*U*_eq_	Occ. (<1)
Cu1	1.000000	1.000000	1.000000	0.0141 (2)	
K1	0.53561 (17)	0.08774 (15)	0.80051 (12)	0.0182 (2)	
O1	1.1046 (5)	0.7828 (4)	0.9188 (4)	0.0139 (6)	
O2	0.6795 (5)	0.7931 (5)	0.9232 (4)	0.0139 (6)	
O3	0.9423 (5)	0.4577 (5)	0.7685 (4)	0.0166 (7)	
O4	0.5056 (5)	0.4613 (4)	0.7870 (4)	0.0168 (7)	
O5	0.1518 (5)	0.2387 (4)	0.4512 (4)	0.0174 (7)	
O6	−0.0303 (6)	−0.0777 (5)	0.2817 (4)	0.0194 (7)	
O7A	0.3910 (18)	−0.2142 (15)	0.5335 (14)	0.028 (2)	0.5
O7B	0.3555 (18)	−0.3016 (16)	0.5653 (14)	0.027 (2)	0.5
C1	0.9305 (7)	0.6178 (6)	0.8431 (5)	0.0125 (8)	
C2	0.6818 (7)	0.6206 (6)	0.8495 (5)	0.0129 (8)	
C3	0.0359 (7)	0.0482 (6)	0.4216 (6)	0.0138 (8)	
Li1	0.1867 (13)	0.3767 (13)	0.6931 (10)	0.0205 (17)	
H1	0.481 (15)	−0.288 (12)	0.557 (10)	0.06 (3)*	
H2	0.283 (15)	−0.343 (13)	0.491 (11)	0.07 (3)*	

### Poly[dipotassium [tri-µ-oxalatocopper(II)dilithium] dihydrate] (III) Atomic displacement parameters (Å^2^)

**Table:**

	*U*^11^	*U*^22^	*U*^33^	*U*^12^	*U*^13^	*U*^23^
Cu1	0.0105 (4)	0.0100 (4)	0.0191 (4)	0.0037 (3)	0.0009 (3)	−0.0015 (3)
K1	0.0195 (5)	0.0173 (5)	0.0177 (5)	0.0079 (4)	0.0026 (4)	0.0031 (4)
O1	0.0106 (14)	0.0118 (14)	0.0176 (16)	0.0044 (11)	0.0000 (11)	0.0000 (12)
O2	0.0119 (14)	0.0156 (15)	0.0133 (15)	0.0064 (12)	0.0018 (11)	−0.0001 (12)
O3	0.0171 (15)	0.0116 (14)	0.0204 (17)	0.0064 (12)	0.0047 (13)	0.0005 (12)
O4	0.0108 (14)	0.0102 (14)	0.0229 (17)	0.0003 (12)	−0.0022 (12)	−0.0014 (12)
O5	0.0223 (16)	0.0086 (14)	0.0159 (16)	0.0012 (12)	0.0031 (13)	0.0018 (12)
O6	0.0236 (17)	0.0182 (16)	0.0121 (16)	0.0051 (13)	0.0001 (13)	0.0014 (13)
O7A	0.026 (5)	0.012 (4)	0.043 (6)	0.012 (4)	−0.003 (4)	−0.011 (4)
O7B	0.019 (5)	0.014 (5)	0.036 (6)	0.007 (4)	−0.009 (4)	−0.017 (4)
C1	0.014 (2)	0.0117 (19)	0.013 (2)	0.0048 (16)	0.0038 (16)	0.0050 (16)
C2	0.013 (2)	0.016 (2)	0.0092 (19)	0.0061 (17)	0.0011 (15)	0.0027 (16)
C3	0.016 (2)	0.011 (2)	0.017 (2)	0.0089 (17)	0.0019 (17)	0.0034 (17)
Li1	0.010 (3)	0.029 (4)	0.021 (4)	0.010 (3)	0.000 (3)	−0.002 (3)

### Poly[dipotassium [tri-µ-oxalatocopper(II)dilithium] dihydrate] (III) Geometric parameters (Å, º)

**Table:**

Cu1—O2	1.938 (3)	O2—C2	1.284 (5)
Cu1—O2^i^	1.938 (3)	O3—C1	1.233 (5)
Cu1—O1^i^	1.942 (3)	O3—Li1^viii^	1.907 (8)
Cu1—O1	1.942 (3)	O4—C2	1.230 (5)
K1—O7A	2.609 (10)	O4—Li1	1.901 (8)
K1—O4	2.814 (3)	O5—C3	1.245 (5)
K1—O2^ii^	2.821 (3)	O5—Li1	1.997 (9)
K1—O7B	2.854 (10)	O6—C3	1.255 (5)
K1—O1^iii^	2.878 (3)	O6—Li1^vii^	2.049 (9)
K1—O3	2.919 (3)	O7A—H1	0.95 (8)
K1—O2^iv^	2.946 (3)	O7A—H2	0.89 (9)
K1—O1^v^	3.036 (3)	O7B—H1	0.75 (8)
K1—O7A^vi^	3.039 (12)	O7B—H2	0.68 (9)
K1—O6^vii^	3.146 (3)	C1—C2	1.549 (6)
K1—O6^vi^	3.178 (3)	C3—C3^vii^	1.571 (9)
O1—C1	1.271 (5)		
			
O2—Cu1—O2^i^	180.0	O1^iii^—K1—O6^vi^	63.34 (8)
O2—Cu1—O1^i^	93.40 (12)	O3—K1—O6^vi^	58.18 (8)
O2^i^—Cu1—O1^i^	86.60 (12)	O2^iv^—K1—O6^vi^	61.26 (8)
O2—Cu1—O1	86.60 (12)	O1^v^—K1—O6^vi^	131.85 (9)
O2^i^—Cu1—O1	93.40 (12)	O7A^vi^—K1—O6^vi^	75.66 (19)
O1^i^—Cu1—O1	180.00 (9)	O6^vii^—K1—O6^vi^	155.90 (12)
O7A—K1—O4	119.6 (2)	C1—O1—Cu1	110.8 (3)
O7A—K1—O2^ii^	134.7 (2)	C1—O1—K1^iii^	137.7 (3)
O4—K1—O2^ii^	70.35 (9)	Cu1—O1—K1^iii^	94.37 (11)
O7A—K1—O7B	13.6 (3)	C1—O1—K1^ix^	133.0 (3)
O4—K1—O7B	131.3 (3)	Cu1—O1—K1^ix^	90.46 (11)
O2^ii^—K1—O7B	127.0 (2)	K1^iii^—O1—K1^ix^	77.56 (8)
O7A—K1—O1^iii^	133.6 (2)	C2—O2—Cu1	110.7 (3)
O4—K1—O1^iii^	101.72 (9)	C2—O2—K1^ii^	133.6 (3)
O2^ii^—K1—O1^iii^	76.43 (9)	Cu1—O2—K1^ii^	97.23 (11)
O7B—K1—O1^iii^	125.6 (2)	C2—O2—K1^x^	132.4 (3)
O7A—K1—O3	114.9 (3)	Cu1—O2—K1^x^	92.37 (11)
O4—K1—O3	56.61 (8)	K1^ii^—O2—K1^x^	79.95 (8)
O2^ii^—K1—O3	106.84 (9)	C1—O3—Li1^viii^	136.6 (4)
O7B—K1—O3	125.5 (2)	C1—O3—K1	112.5 (3)
O1^iii^—K1—O3	70.04 (9)	Li1^viii^—O3—K1	107.9 (3)
O7A—K1—O2^iv^	80.2 (2)	C2—O4—Li1	139.7 (4)
O4—K1—O2^iv^	159.58 (9)	C2—O4—K1	116.4 (3)
O2^ii^—K1—O2^iv^	100.05 (8)	Li1—O4—K1	103.7 (3)
O7B—K1—O2^iv^	69.0 (2)	C3—O5—Li1	113.3 (4)
O1^iii^—K1—O2^iv^	57.99 (8)	C3—O6—Li1^vii^	111.7 (4)
O3—K1—O2^iv^	112.41 (9)	C3—O6—K1^vii^	100.4 (3)
O7A—K1—O1^v^	80.5 (3)	Li1^vii^—O6—K1^vii^	89.8 (2)
O4—K1—O1^v^	113.85 (9)	C3—O6—K1^vi^	99.4 (3)
O2^ii^—K1—O1^v^	57.50 (8)	Li1^vii^—O6—K1^vi^	95.6 (2)
O7B—K1—O1^v^	70.1 (2)	K1^vii^—O6—K1^vi^	155.90 (12)
O1^iii^—K1—O1^v^	102.44 (8)	K1—O7A—K1^vi^	115.8 (4)
O3—K1—O1^v^	164.28 (9)	K1—O7A—H1	100 (5)
O2^iv^—K1—O1^v^	72.19 (8)	K1^vi^—O7A—H1	113 (5)
O7A—K1—O7A^vi^	64.2 (4)	K1—O7A—H2	144 (6)
O4—K1—O7A^vi^	64.04 (18)	K1^vi^—O7A—H2	96 (6)
O2^ii^—K1—O7A^vi^	132.00 (18)	H1—O7A—H2	81 (7)
O7B—K1—O7A^vi^	77.7 (4)	K1—O7B—H1	89 (6)
O1^iii^—K1—O7A^vi^	126.0 (2)	K1—O7B—H2	135 (8)
O3—K1—O7A^vi^	58.7 (2)	H1—O7B—H2	113 (9)
O2^iv^—K1—O7A^vi^	127.94 (18)	O3—C1—O1	126.1 (4)
O1^v^—K1—O7A^vi^	131.4 (2)	O3—C1—C2	118.0 (4)
O7A—K1—O6^vii^	82.2 (2)	O1—C1—C2	115.9 (4)
O4—K1—O6^vii^	61.81 (9)	O4—C2—O2	125.6 (4)
O2^ii^—K1—O6^vii^	63.59 (8)	O4—C2—C1	118.7 (4)
O7B—K1—O6^vii^	84.8 (2)	O2—C2—C1	115.7 (3)
O1^iii^—K1—O6^vii^	139.77 (9)	O5—C3—O6	128.1 (4)
O3—K1—O6^vii^	116.35 (9)	O5—C3—C3^vii^	116.4 (5)
O2^iv^—K1—O6^vii^	131.16 (9)	O6—C3—C3^vii^	115.6 (5)
O1^v^—K1—O6^vii^	60.12 (8)	O4—Li1—O3^xi^	131.6 (5)
O7A^vi^—K1—O6^vii^	81.9 (2)	O4—Li1—O5	108.5 (4)
O7A—K1—O6^vi^	80.2 (2)	O3^xi^—Li1—O5	117.8 (4)
O4—K1—O6^vi^	114.08 (9)	O4—Li1—O6^vii^	102.2 (4)
O2^ii^—K1—O6^vi^	139.69 (9)	O3^xi^—Li1—O6^vii^	97.4 (4)
O7B—K1—O6^vi^	82.0 (2)	O5—Li1—O6^vii^	82.4 (3)

Symmetry codes: (i) −*x*+2, −*y*+2, −*z*+2; (ii) −*x*+1, −*y*+1, −*z*+2; (iii) −*x*+2, −*y*+1, −*z*+2; (iv) *x*, *y*−1, *z*; (v) *x*−1, *y*−1, *z*; (vi) −*x*+1, −*y*, −*z*+1; (vii) −*x*, −*y*, −*z*+1; (viii) *x*+1, *y*, *z*; (ix) *x*+1, *y*+1, *z*; (x) *x*, *y*+1, *z*; (xi) *x*−1, *y*, *z*.

### Cu—O bond Lengths of Compound III

**Table:**

Bond	Bond distance (Å)
Cu—O1	1.942 (3)
Cu—O1^i^	1.942 (3)
Cu—O2	1.938 (3)
Cu—O2^i^	1.938 (3)
Cu—O6	2.473 (4)
Cu—O6^i^	2.473 (4)

Symmetry codes: (i) -*x* + 2, -*y* + 2, -*z* + 2.
